# Early stage of radiological expertise modulates resting-state local coherence in the inferior temporal lobe

**DOI:** 10.1093/psyrad/kkac024

**Published:** 2022-12-09

**Authors:** Minghao Dong, Peiming Zhang, Weilu Chai, Xiaoyan Zhang, Bihong T Chen, Hongmei Wang, Jia Wu, Chao Chen, Yi Niu, Jimin Liang, Guangming Shi, Chenwang Jin

**Affiliations:** Engineering Research Center of Molecular and Neuro Imaging of Ministry of Education, School of Life Science and Technology, Xidian University, Xi’an City, Shaanxi 710071, China; Xian Key Laboratory of Intelligent Sensing and Regulation of tran-Scale Life Information, Xi’an City, Shaanxi 710071, China; Key Laboratory of Intelligent Perception and Image Understanding of Ministry of Education, School of Artificial Intelligence, Xidian University, Xi’an City, Shaanxi 710071, China; Engineering Research Center of Molecular and Neuro Imaging of Ministry of Education, School of Life Science and Technology, Xidian University, Xi’an City, Shaanxi 710071, China; Key Laboratory of Intelligent Perception and Image Understanding of Ministry of Education, School of Artificial Intelligence, Xidian University, Xi’an City, Shaanxi 710071, China; Engineering Research Center of Molecular and Neuro Imaging of Ministry of Education, School of Life Science and Technology, Xidian University, Xi’an City, Shaanxi 710071, China; City of Hope Medical Center, Duarte City, California 91010, USA; Department of Medical Imaging, First Affiliated Hospital of Medical College, Xi’an Jiaotong University, Xi’an City, Shaanxi 710000, China; School of Foreign Languages, Northwestern Polytechnical University, Xi'an City, Shaanxi 710071, China; PLA Funding Payment Center, Beijing 100000, China; Key Laboratory of Intelligent Perception and Image Understanding of Ministry of Education, School of Artificial Intelligence, Xidian University, Xi’an City, Shaanxi 710071, China; School of Electronics and Engineering, Xidian University, Xi'an City, Shaanxi 710071, China; Key Laboratory of Intelligent Perception and Image Understanding of Ministry of Education, School of Artificial Intelligence, Xidian University, Xi’an City, Shaanxi 710071, China; Department of Medical Imaging, First Affiliated Hospital of Medical College, Xi’an Jiaotong University, Xi’an City, Shaanxi 710000, China

**Keywords:** visual expertise, resting state fMRI, radiologists, inferior temporal cortex

## Abstract

**Background:**

The visual system and its inherent functions undergo experience-dependent changes through the lifespan, enabling acquisition of new skills. Previous fMRI studies using tasks reported increased specialization in a number of cortical regions subserving visual expertise. Although ample studies focused on representation of long-term visual expertise in the brain, i.e. in terms of year, monthly-based early-stage representation of visual expertise remains unstudied. Given that spontaneous neuronal oscillations actively encode previous experience, we propose brain representations in the resting state is fundamentally important.

**Objective:**

The current study aimed to investigate how monthly-based early-stage visual expertise are represented in the resting state using the expertise model of radiologists.

**Methods:**

In particular, we investigated the altered local clustering pattern of spontaneous brain activity using regional homogeneity (ReHo). A cohort group of radiology interns (n = 22) after one-month training in X-ray department and matched laypersons (n = 22) were recruited after rigorous behavioral assessment.

**Results:**

The results showed higher ReHo in the right hippocampus (HIP) and the right ventral anterior temporal lobe (vATL) (corrected by Alphasim correction, *P < 0.05*). Moreover, ReHo in the right HIP correlated with the number of cases reviewed during intern radiologists’ training (corrected by Alphasim correction, *P < 0.05*).

**Conclusions:**

In sum, our results demonstrated that the early stage of visual expertise is more concerned with stabilizing visual feature and domain-specific knowledge into long-term memory. The results provided novel evidence regarding how early-stage visual expertise is represented in the resting brain, which help further elaborate how human visual expertise is acquired. We propose that our current study may provide novel ideas for developing new training protocols in medical schools.

## Introduction

For many years, radiology has been the most common clinical specialty (Krupinski, 2010), where an average of 120 medical image perception events take place each second (Krupinski, 2010). Radiological images are not self-explainary, therefore, medical imaging interpretation is indispensable in diagnostic process (Bankman, 2008). It lies in radiologists’ exceptional radiological-specific visual recognition skill, which enables the identification of pathological regions, which are visually homogenous, to render diagnosis. This perceptual specialty, i.e. visual expertise, is acquired through long-term training after reviewing at least hundreds of cases (Nodine *et al*., 2002; Krupinski and Samei, 2010).

The brain and its inherent functions undergo experience-dependent changes through the lifespan, enabling acquisition of new skills (Dong *et al*., 2014; Li, 2016). Adaptive changes are seen in acquisition of expertise in visual object recognition, i.e. fine level visual discrimination of homogeneous stimuli (Harel, 2016; Wang *et al*., 2021). The visual expertise is gradually acquired through cumulative experience within a given object category (Krupinski, 2010; Song *et al*., 2022; Zhang *et al*., 2022). Continuing effort has been expanded to better understand the neural substrate underlying such long-term expertise (Haller and Radue, 2005; Manning *et al*., 2005; Harley *et al*., 2009; Bilalić *et al*., 2014; Ouellette *et al*., 2020). Haller et al. (Haller and Radue, 2005) and Ouellette et al. (Ouellette *et al*., 2020) observed differentiated patterns of activations in the inferior temporal lobe between radiologists and novices. Harley et al. specifically investigated the visual pathway and reported engagement of the inferior temporal lobe when radiologists detected abnormalities in chest radiographs (Harley *et al*., 2009). In sum, experience in visual recognition alters brain specialty as a hierarchical movement from low-level visual representations to higher level categorical organization of conceptual representations in coordination with updated representations in the memory system. Although ample studies focused on representation of long-term expertise in the brain, i.e. in terms of year, monthly-based early-stage representation of visual expertise remains unstudied. Given that studies from behavioral accounts suggested that radiologists who interpreted a high volume of mammograms (2500∼4000) annually did show distinct behavioral patterns as compared with low-volume physicians (Smith-Bindman *et al*., 2005), we postulate that early stage of visual expertise should explicate different pattern of central representations.

Apart from attention to brain responses to tasks, we propose that the information embedded in the intrinsic brain activity, i.e. resting state data, is also important, because the spontaneous low-frequency fluctuations in the restful brain is involved in the coding of previous experience (Miall and Robertson, 2006; Dougherty *et al*., 2007). Moreover, experience-dependent neuroplastic changes shape the pattern of spontaneous activity within the resting brain (Albert *et al*., 2009; Thomas and Baker, 2012) and such alterations bear behavior significance (Barkhof *et al*., 2014; Dong *et al*., 2014; Kelly and Castellanos, 2014). Accordingly, a small but emerging body of literature has shown that resting state brain activity is a new window to understand the neural substrate of expertise in the context of neural plasticity (Barkhof *et al*., 2014).

Available evidence has elucidated that visual expertise is supported by increased specialization in a number of cortical regions involved in domain-specific tasks. In the current, we investigated how this regional specialization is represented in the resting brain. Previous studies used the regional homogeneity (ReHo) as the reliable and replicable metric of local brain coherence in the resting state (Dong *et al*., 2015; Jin *et al*., 2018). Accordingly, in the current study, we evaluated the ReHo differences between a group of radiology interns (RIG) (N = 22) and a group of matched healthy layer-person (HCG) to assess how early-stage radiological expertise alters local brain coherence in the resting sate. A set of behavioral tasks were employed to assess the level of radiological expertise and visual expertise in other domains (Zhang *et al*., 2022). Given that studies from cognitive accounts showed that low-volume readers is featured as matching the newly acquired radiologic scene to previously learned radiologic patterns or templates representing normal anatomic variants, while high-volume readers tends to resemble automated procedures (Krupinski and Samei, 2010), we expected to see changes in the brain regions supportive of associative memory. Furthermore, we examined how radiological experience was related to the level of local brain coherence in radiologists. We proposed that our study provides the first evidence on how early-stage visual expertise changes the brain representation in the resting state.

## Methods

All research procedures were approved by the Ethical Committee of *First Affiliated Hospital of Medical College, Xian Jiaotong University* subcommittee on Human Studies and were conducted in accordance with the Declaration of Helsinki. Written informed consent was obtained from the participants after the experimental procedures were fully explained.

### Experimental procedure

A group of radiology interns (RIG) and a group of matched non-medical healthy normal control group (HCG) were recruited in the current study. First, RIG consisted of interns from radiology department of *First Affiliated Hospital of Medical College, Xian Jiaotong University*. Specifically, the interns undertook a month-long training in X-ray department followed by duty rotations in other departments, such as MRI, B-scan ultrasonography, positron emission tomography-computed tomography (PET-CT) in a span of 4 months. For the participants prescreening procedure, handedness (Oldfield, 1971), level of face expertise (Duchaine and Nakayama, 2006) and visual expertise in other domains (e.g. cars, chess, birds and mushrooms) were eliminated using questionaries, tasks and face-to-face interview. Then, the matched control subjects were selected from the Control Subjects Database for Visual Expertise (CSDVE) set up by our group for visual expertise studies. Note that subjects from HCE had no experience in radiography and visual expertise in domains of cars, chess, birds and mushrooms. Scores of handedness, level of education and level of face expertise were also collected in the CSDVE. Second, MRI scanning was conducted without informing the purpose of this study (*elaborated in 2.4*), and was immediately followed by behavior measurement conduced in a sperate room (*elaborated in 2.3*) including Radiological Expertise Task (RET) and Cambridge Face Memory Test (CFMT) (*elaborated in 2.3*). Please note that the scores for the CFMT in the CSDVE were only used for subject selection, and the results of the CFMT after MRI scanning were included for data analysis.

### Subjects

In the current study, 22 subjects in the RIG (11 males, mean age 23 ± 0.7 years (mean ± standard deviation, SD)) and 22 matched subjects in the NCG (11 males, mean age 23 ± 0.5 years (mean ± SD)) were recruited. The two groups were matched for sex, level of education and age. Specifically, the subjects in the RIG were undergraduate medical students from national medical schools following the same training protocol. They underwent four-week training in the X-ray department. In the training, they reviewed 25–35 cases each day per person for a duration of 26 ± 2.5 (mean ± SD) days. For each case, the radiology interns were required to identify the pathologies in the X-ray films displayed on the monitor screen and filed medical report for each case. Therefore, their experience was centered on visual recognition in interpreting X-ray images. The tutors gave written feedback based on each medical report. Each case report of the radiology interns is matched for ‘degree of agreement’ against the decision of the tutor radiologist. In total, their visual expertise in radiological images were built and gradually reinforced with experience of a minimum of 600 cases as recorded in the Picture Archiving and Communication System (PACS) during duty rotation.

Subjects from both groups reported no past or current neurological disorders, neuropsychological disorders or psychiatric disorders and did not take drugs or illegal medication before or during the study. All the participants gave informed consent and had normal and corrected-to-normal vision when participating in tests outside the scanner.

### Behavior measurement

**Figure 1:**
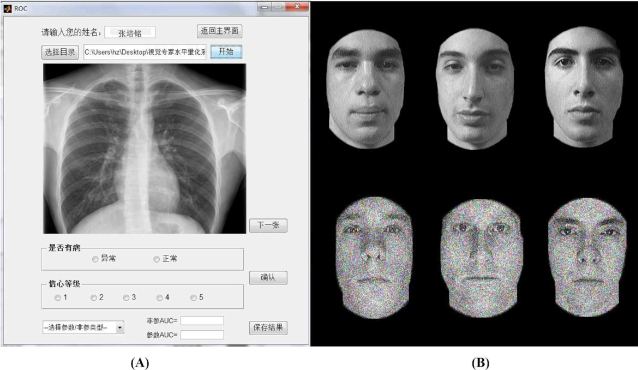
Samples of behavioral tests. (A) stimulus used in Cambridge Face Memory Test; (B) software interface for the Radiological Expertise Task.

The behavioral tests were undertaken after MRI data collection to eliminate the influence of tasks on the resting state. Basically, The level of face expertise was evaluated using the Cambridge Face Memory Test (CFMT) (Duchaine and Nakayama, 2006), a standardized test of face recognition (Fig. 1A). The level of perceptual expertise in X-ray images was evaluated by the Radiological Expertise Task (Fig. 1B). The RET was performed on in-house software developed by our group (Chinese Software Patent NO. 2018SR036699, http://rsvp.dingdongyun.com/). The experimental procedure and algorithms in RET are under guidance of *The Handbook of Medical Image Perception and Techniques* (Bankman, 2008), which is considered as a standardized test of radiological expertise (Metz, 2006; Krupinski and Samei, 2010). Please note that the technical details were provided in our previous publication (Wang *et al*., 2021).

### MRI data acquisition

A 3Telsa MRI system (EXCITE, General Electric, Milwaukee, Wisc.) was utilized in MRI scanning. A standard birdcage head coil was used, along with restraining foam pads to minimize head motion and to diminish scanner noise. The scanning was conducted in the *First Affiliated Hospital of Medical College, Xi'an Jiaotong University*, including a resting scan, a localizer scan, a structural and a DTI scan. To eliminate the time-of-day effect, the scanning was performed from 9:00 a.m. to 11:30 a.m (Murray *et al*., 2009; Hasler *et al*., 2014). The localizer data and DTI data were used for other studies and therefore not reported in this study.

During the resting scan, subjects were instructed not to keep their mind blank and keep their eyes open in the entire session. For the resting scan, whole brain images were acquired with a gradient-echo single-shot echo planar imaging sequence using the following parameters: matrix = 64 × 64, field of view (FOV) = 240 mm; repetition time (TR) = 2 s; echo time (TE) = 30 ms. Thirty-two interleaved axial slices were oriented parallel to each participant's anterior commissure-posterior commissure (AC-PC) line, voxel size = 3.8 × 3.8 × 5.0 mm, no gap. The resting-state fMRI scans lasted for 6 minutes and 20 second (Somandepalli *et al*., 2015), resulting in 190 volumes. Additionally, an MPRAGE T1-magnetization high resolution anatomical image (1 × 1 × 1 mm resolution) was also acquired for each participant for co-registration and display of the DTI fiber bundle data with the following parameters: echo time (TE) = 2.26 ms, repetition time (TR) = 1900 ms, flip angle = 9^o^, field of view (FOV) = 256 mm, and matrix = 256 × 256. This yielded 176 contiguous 1 mm thick slices in the sagittal orientation. The structural images were used to exclude potential clinical abnormalities by two expert radiologists. No participants were excluded at this level.

### Functional data preprocessing

Statistical Parametric Mapping (SPM12) (http://www.fil.ion.ucl.ac.uk/spm) and Data Processing Assistant for Resting-State fMRI (DPARSF) V2.4 advanced edition (http://www.restfmri.net/forum/DPARSF) (Chao-Gan and Yu-Feng, 2010) was used in the data preprocessing procedures under MATLAB2009a.

#### Resting data processing

The first 10 volumes in each subject were discarded to eliminate non-equilibrium effects of magnetization. The data preprocessing included temporal correction for acquisition delay between slices, motion correction, co-registration to the subject's anatomical images in native space, normalization to the using the deformation field maps obtained from structural image segmentation, and the segmentation routine in Statistical Parameter Mapping 12 (SPM12). No head motions were found exceeding 1 mm of movement or 1° of rotation in any direction. The normalized images were resampled to 3 mm isotropic voxels, which were then spatially smoothed with a 6 mm full width at half maximum Gaussian kernel. Lastly, the linear trend was removed and temporal filtering (0.01–0.08 Hz (Biswal *et al*., 1995; Lowe *et al*., 1998)) were performed on the time series of each voxel to reduce the effect of low-frequency drifts and high-frequency noise.

#### ReHo map generation

Regional homogeneity (ReHo) was used to assess local coherence of spontaneous brain activity in the resting state. The ReHo was obtained using the REST package (http://resting-fmri.sourceforge.net) (Yang *et al*., 2007). In brief, fast Fourier transform (FFT) was used to preprocessed transform time series of each voxel to the frequency domain. Given that the power of a given frequency was proportional to the square of the amplitude of this frequency component, the square root was calculated at each frequency of the power spectrum and the averaged square root was obtained across 0.01–0.08Hz at each voxel. This averaged square root was taken as the REHO ReHo for each voxel. For standardization, the ReHo of each voxel was divided by the global mean ReHo value for each subject, resulting in a relative ReHo (Raichle *et al*., 2001).

### Statistical analysis

#### Inter-group ReHo analysis

Statistical analysis was performed in SPM12. Voxel-wise comparison ReHo analysis was conducted across the whole brain. For the Inter-group analysis, a two-sample t-test was performed to detect the ReHo difference between the two groups. The results were considered significant above the threshold of *P < 0.05*, corrected for the multiple correction at the cluster level, with an underlying threshold of *P < 0.00*1 uncorrected at the voxel level. For multiple comparisons, Monte Carlo simulations were performed using the AFNI AlphaSim program.

#### Correlation analysis

The voxel-wise *Pearson's correlation analysis* was conducted between ReHo and outcome of behavior tasks, i.e. CMFT, RET, RT of RET, cases reviewed in total, to investigate the relationship between the ReHo and behavior measurements in the RIG. For the level of significance, the threshold of *P < 0.05*, corrected for the multiple correction at the cluster level, with an underlying threshold of *P < 0.001* uncorrected at the voxel level was applied. For multiple comparisons, Monte Carlo simulations were performed using the AFNI AlphaSim program.

## Results

### Results of behavior measurement

**Figure 2:**
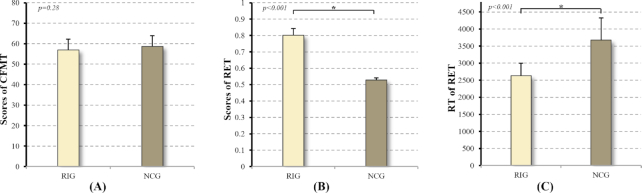
Results of behavior measurement for both groups. (A) The level of perceptual expertise in the domain of faces as measured by the Cambridge Face Memory Test; (B) The level of perceptual expertise in the domain of radiological images as assessed by the Radiological Expertise Task. The radiology interns group had a significantly larger AUC than the normal control group (*P* < 0.001, Mann-Whitney test), indicating better visual recognition ability in radiological images; (C) Response time of both groups in the Radiological Expertise Task. RET: Radiological Expertise Task; RIG: radiology interns group; NECG: normal control group; CMFT: Cambridge Face Memory Test. *Indicates significant group differences (*P* < 0.05).

No statistical difference was found in the level of face expertise, as indexed by the results of Cambridge Face Memory Test (CFMT) between groups (*P = 0.28, Mann-Whitney test*, Table 1, Fig. 2A). The RIG was significantly faster in recognizing chest abnormalities than the control group (*P =* 1.3 × 10^−7^*, Mann-Whitney test*, Table 1, Fig. 2B). Furthermore, the RIG group had significantly higher level of radiological expertise, supported by higher AUC from Radiological Expertise Task (RET) in the RIG than the control group (*P =* 3.8 × 10^−22^*, Mann-Whitney test*, Table 1, Fig. 2C). More importantly, for RIG, the outcome of RET fell within the interval of 0.73–0.86, indicating that the level of behavioral performance approached the level of expertise (Krupinski, 2010). Based on the results, we have to state that response time (RT) of RET did reflect the behavioral expertise, even though this parameter was not used in clinical practice to determine radiologists’ visual expertise. In sum, the visual expertise in radiology images interpretation was obtained through training across reviewing hundreds of cases (Nodine *et al*., 2002; Krupinski, 2010), illustrated as faster and better recognition performance in the training domain of expertise.

**Table 1:** The: results of behavioral measurement between the two groups.

	Radiologists (*n* = 22)		Controls (*n* = 22)	
	Mean	SD	Mean	SD
**face expertise**	56.95	5.23	58.68	5.31
*radiological expertise	0.80	0.04	0.53	0.04
*response time of RET(s)	2.6	0.4	3.7	0.7
**days of training**	26	2.4	N/A	N/A
**cases reviewed**	767.4	82.6	N/A	N/A

*denotes the item that shows significant difference between groups (*P < 0.001*)

^^^denotes that Mann-Whitney test was used

Abbreviations: RET-radiological expertise test; s-seconds; SD-standard deviation.

### Results of inter-group ReHo analysis

**Figure 3:**
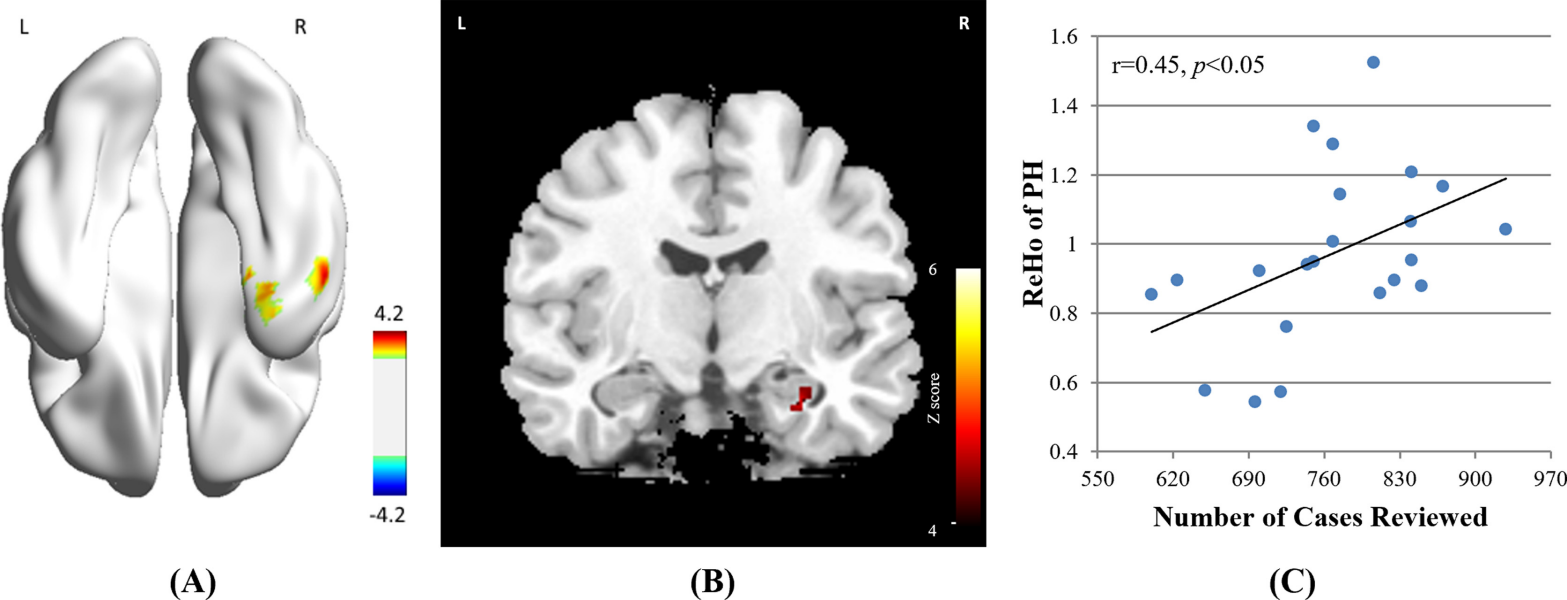
Results of Inter-group ReHo analysis and voxel-wise correlation analysis between ReHo and behavioral measurement. (A) The RIG (n = 22) showed significantly higher ReHo in the right HIP and the right vATL(corrected by Alphasim correction, *P* < 0.05) compared with NCG (n = 22); (B) Significant correlation between ReHo and the number of cases reviewed during duty rotation in RIG was found the in the right HIP (corrected by Alphasim correction, *P* < 0.05); (C) The scatter plot map computed as ReH of the peak voxel (34, −10, −20) in the correlation analysis and the number of cases reviewed. Please note that this map is only for illustration purpose, otherwise there would be the risk of a circular analysis. RIG: radiology intern group; HCG: healthy control group; HIP: hippocampus; vATL: ventral anterior temporal lobe.

Significantly higher ReHo were found for the RIG in the right hippocampus (HIP) and the right ventral anterior temporal lobe (vATL), as the result of the two-sample *t*-test (*P < 0.05, multiple comparison corrected*, Fig. 3A, Table 2). No significant decrement in ReHo were found in any other brain regions.

**Table 2:** Peak: activations of ReHo differences between two groups.

			MNI Coordinates (cluster maxima)				
	Hemisphere	BA	x	y	z	voxels	t (cluster maxima)
**HIP**	R	–	28	−7	−22	67	3.7
**vATL**	R	20	55	−13	−31	108	4.1

Abbreviations: BA: Brodmann Area; HIP: hippocampus; L-left; MNI: Montreal Neurological Institute; R-right; vATL: ventral anterior temporal lobe.

### Results of the correlation analysis

Voxel-wise correlation analysis illustrated a significant positive correlation between the level of local brain coherence in the right HIP of the RIG and the radiological expertise, indexed by the number of cases reviewed in the RIG (*P* < 0.05, *r* = 0.45, Fig. 3B and C, Table 3). The MNI coordinate of peak voxel is (34, −10, 20). No such correlations or anticorrelations were found between outcomes of other behavior tests and ReHo in RIG and NCG.

**Table 3:** Significant: voxel-wise correlation between ReHo of HIP and the number of cases reviewed by RIG (*n* = 22).

		MNI Coordinates (cluster maxima)				
	Hemisphere	x	y	z	Voxels	Z (cluster maxima)
**HIP**	R	34	−10	−20	21	4.5

Abbreviations: HIP: hippocampus; R: right.

## Discussion

The model of radiologists serves as a singular but important model to study visual expertise due to their exceptional visual recognition skill. Previous neuroimaging studies centered on neuronal response under tasks, however, our current study focused on the restful brain by investigating a much ignored issue of how radiological experience or expertise alters the level of local brain coherence in expertise-related brain regions. Specifically, a group of radiography interns were recruited after one month duty rotation in X-ray department. The results of the behavior data analysis showed that the radiography interns group (RIG) significantly outperformed the healthy control group (HCG) in radiological visual recognition tasks (Fig. 1 and Table 1), which echoed previous findings. The results of imaging data analysis showed higher local brain coherence, as indexed by ReHo, in the right hippocampus (HIP) and the right vATL (*P < 0.05, AlphaSim correction*, Fig. 3A, Table 2). Moreover, voxel-wise correlation analysis demonstrated that ReHo in the right HIP correlated with the number of cases reviewed during intern radiologists’ training (*P < 0.05, AlphaSim correction*, Fig. 3B, C, Table 3). We propose that the RIG used in our study is unique, because it helps to understand the early stage of visual expertise, which has never been studied before. The results provided novel evidence regarding how early-stage visual expertise is represented in the resting brain, which help further elaborate how human visual expertise is acquired. We propose that our current study may provide new ideas for developing new training protocols in medical schools.

### Increased local coherence of HIP and vATL in intern radiologists

Our results elucidated higher ReHo in the right hippocampus (HIP) in RIG (Fig. 3A, Table 2). The HIP is constantly reported in expert object recognition for radiologists (Haller and Radue, 2005), car experts (Harel *et al*., 2010) and chess players (Hänggi *et al*., 2014). HIP is a regarded as a core region in memory system, responsible for knowledge storage (Woollett and Maguire, 2009). The chunking theory, i.e. template theory, proposes that experts store domain-specific perceptual patterns, namely chunks in their long-term memory during study and practice (Goldin, 1979). The acquisition of radiological expertise involves working through a large number of cases, which then form a ‘database’ that serve as anchors for diagnostic hypothesis testing. This database consists of domain-specific perceptual patterns, i.e. chunks, stored in long-term memory, which can be used as basic units of meaning. Particularly, HIP is preferentially involved in associative memory (Ren *et al*., 2020), which is defined as the ability to learn and remember the relationship between unrelated items (Wang *et al*., 2014). After the duty rotation started, intern radiologists access chunks of domain-specific information in long-term memory. As their practice during rotation becomes continuous and the number of reviewed cases increased, some of the chunks evolve into templates (Gobet and Simon, 1996). Moreover, our results showed that the total number of cases reviewed during their rotation training correlated with the ReHo of HIP (Fig. 3B and C, Table 3). This indicates that the HIP difference between healthy controls and intern radiologists is likely to be affected by intensive learning experience with radiological image interpretation. Also, given that the hippocampus has also been associated with active maintenance of novel information (Ranganath and D'Esposito, 2001), we suggest that the engagement of HIP may be related to the acquisition, storage, update and application of these chunks and templates.

In addition, in the current study, higher ReHo in the RIG was found in the right vATL than in the NCG. This region is reported in visual expertise domain of faces (Kriegeskorte *et al*., 2007), cars (Harel *et al*., 2010) and chess (Hänggi *et al*., 2014). It is a key region for the representation of semantic knowledge (Rice *et al*., 2015) and is involved in lexical access and semantic retrieval (Binder and Desai, 2011). In visual neuroscience, merging of perceptual and conceptual knowledge happens in the vATL (Conway, 2018). As for RIG, in the beginning stage of training, perceptual patterns stored in long-term memory system of inter radiologists is represented without any reference to the actual context of the image. As their case-reading experience accumulates, they are able to place information in context, which is supported by associations between the relevance of the perceived clinical features and stored knowledge. These associations are established through various forms of feedback in a question/answer format in their rotation, such as assigning names to pathological features, discussing convoluted descriptions of clinical features to ion to colleagues, teaching colleagues, puzzling out difficult cases, and participating in research both as subject and as investigator. The associations are represented as domain-specific semantic knowledge, which facilitates the evolvement of perceptual patterns into more complex structures, namely templates, consisting of core information supplemented with slots in which more perceptual or abstract information can be added (Gobet and Simon, 1996). These chunks and templates are stored in long-term memory as the building blocks of visual expertise.

As skill develops, the initial effortful performance is replaced by more automated procedures, indicated by shorter response time (Fig. 2A, Table 1). This efficiency in behavior can be explained by neural-efficiency hypothesis (Neubauer *et al*., 2009), which indicates that experts require fewer brain resources because of practice and automatization of procedures (Grabner *et al*., 2006). Regularly, neurons that ‘fire together, wire together’, where an increase in synaptic efficacy is the outcome of one cell's repeated and persistent stimulation of another cell (Hebb, 2005). Such increased synaptic connectivity may lead to increased local synchronization, as indexed by regional homogeneity in a given brain area (Dong *et al*., 2014). Therefore, we speculate that increased local coherence in the right hippocampus (HIP) and right vATL observed in the RIG in our study may reflect high efficiency of information transfer for low wiring costs due to neuroplasticity modulated by expertise in a specific task. But note that, previous expertise studies reported lower activation or less activated regions (Kelly and Garavan, 2005). Given the increased local coherence in expertise-related areas found in different expertise models form our group (Dong *et al*., 2014), we propose that the effect of expertise is likely to show dissociation between tasks and resting state.

### Significance of intern radiologists for understanding visual expertise

Radiologists are regarded as an important model to study visual expertise by researchers from behavioral (Krupinski and Samei, 2010), cognitive (Kundel *et al*., 2008) and neuroimaging communities (Haller and Radue, 2005). Basically, fine level of visual recognitive skill is the basis of radiologists’ daily medical practice, which enables the identification of pathological regions to render diagnosis from a set of visually similar images (Zhang *et al*., 2022). In our study, RIG consists of intern radiologists after one-month rotation in X-ray department, when they were required to identify the pathologies in the X-ray films displayed on the monitor screen and file medical report for each case. Accordingly, experience of our subjects was centered on visual recognition in interpreting X-ray images. Normally, this perceptual specialty is acquired through intensive training after a minimum of hundreds of cases are reviewed (Nodine *et al*., 2002; Krupinski, 2010). Accordingly, subjects from RIG reviewed at least 767 cases during their rotation (Fig. 2A, Table 1) and the performance reached expertise level demonstrated by the results of Radiological Expertise Task (RET) (Fig. 2B, Table 1). Therefore, the intern radiologists in the current study have acquired visual expertise in the domain of radiology. Nevertheless, we have to point out that behavioral account of studies on radiological expertise did report distinction in diagnostic performance and behavioral patterns between radiological interns and more experienced radiologists (Mallett *et al*., 2014; Bertram *et al*., 2016). Moreover, evidence from cognitive account suggest that performance in the initial state of expertise is more effortful, whereas the performance in the final stages resemble automated procedures (Krupinski and Samei, 2010; Bilalić, 2018). Based on our current findings, we argue that the early stage of visual expertise engages more processes of stabilizing and integrating labile memory traces of object-concept association into long-term memory. Nevertheless, for the end stage of visual expertise, object-concept associations are already learned to criterion and retrieval of domain-specific knowledge is more concerned while process of consolidation and storage of conceptual knowledge is less involved.

However, we have to admit the differences between the early and final stage of visual expertise are invariably relative rather than absolute. In the gradient of visual expertise, the intern radiologists in the current study captured the innate early-stage signatures of this continuum. Our study shed new light on the development of visual expertise.

### Limitation

Several limitations should be taken into consideration when interpreting findings from the current study. Second, the sample size was comparatively small in the current study. In this study, we managed to align all the participants’ training arrangement to starting from the X-ray department to insure homogeneity of expertise. Given the limited number of radiology interns each year, this sample size is the best we were able to achieve. Third, the level of conceptual knowledge in radiological expertise was not measured. There is no standardize behavioral tasks measuring nonvisual knowledge about radiology up to now, however, this is feasible in other domain of visual expertise (Van Gulick *et al*., 2016).

## Conclusion

Our current study provides the first evidence of how early stage of visual experience/expertise modulates resting state local brain coherence in the temporal lobe using the visual expertise model of radiologists. Our results indicates that, as for the early stage of visual expertise, stabilizing and integrating pathological features with domain-specific knowledge into long-term memory is a key process, where HIP, together with vATL, are responsible for updating representations of object templates and forming associations between reviewed samples and conceptual knowledge. We suggest that our study may shed light on the development of visual recognition skills in medical image interpretation. We hope that by revealing the neural mechanism of radiological visual expertise, more efficient education strategies can be developed (McLoud, 2010).

## Abbreviations

AC-PC: anterior commissure-posterior commissure; vATL: ventral anterior temporal lobe; AUC: area under curve; BOLD: blood oxygen level-dependent; CFMT: Cambridge Face Memory Test; DPARSF: Data Processing Assistant for Resting-State fMRI; FFT: fast Fourier transform; fMRI: functional Magnetic Resonance Imaging; FOV: field of view; HC: the hippocampus cortex; HCG: non-medical healthy control group; HIP: hippocampus; PACS: Picture Archiving and Communication System; ReHo: regional homogeneity; RET: Radiological Expertise Task; RIG: radiology interns group; ROC: Receiver operating characteristic; SPM: Statistical Parameter Mapping; TE: echo time; TR: repetition time.
